# MARITAL DISSOLUTION AND COGNITIVE FUNCTION IN MID- AND LATE ADULTHOOD: FOCUS ON WITHIN-PERSON EFFECTS AND AND TIMING

**DOI:** 10.1093/geroni/igae098.2097

**Published:** 2024-12-31

**Authors:** Julia Tucker, Mateo Farina, Sae Hwang Han

**Affiliations:** The University of Texas Austin, Austin, Texas, United States; University of Texas at Austin, Austin, Texas, United States; University of Texas at Austin, Austin, Texas, United States

## Abstract

There is a growing literature documenting cognitive health disparities between married individuals and their widowed and divorced counterparts in middle and later adulthood. However, how cognitive function changes within individuals as they experience widowhood and divorce transitions, and whether these changes vary depending on the timing of the marital dissolution, remain understudied. Framed within the life course perspective, this study aimed to examine within–person changes in cognitive function associated with widowhood and divorce transitions in mid– and later life, respectively. We used 11 waves of longitudinal data from the Health and Retirement Study (1998–2018; 121,505 person–wave observations collected from 20,114 persons). Cognitive function was evaluated using a modified version of the Telephone Interview for Cognitive Status (range: 0–27). Multilevel models using person–mean centering were employed to estimate within–person associations between the two forms of marital transitions and subsequent cognitive function. Results indicated that widowhood transitions were associated with within-person changes in cognitive function for women, but in the opposite direction depending on the timing of the transition: midlife widowhood transitions (between ages 51 and 64) were linked to worse cognitive function, whereas late-life widowhood transitions (age 65+) were associated with better cognitive function. Widowhood transitions were unrelated to cognitive function for men, and divorce transitions were unrelated to changes in cognitive function for men or women. These results underscore the heterogeneity in the association between marital transitions and cognitive function, which vary by gender, life course stage, and the nature of marital dissolution.

